# Climate based multi-year predictions of the Barents Sea cod stock

**DOI:** 10.1371/journal.pone.0206319

**Published:** 2018-10-24

**Authors:** Marius Årthun, Bjarte Bogstad, Ute Daewel, Noel S. Keenlyside, Anne Britt Sandø, Corinna Schrum, Geir Ottersen

**Affiliations:** 1 Geophysical Institute, University of Bergen, 5007 Bergen, Norway; 2 Bjerknes Centre for Climate Research, 5020 Bergen, Norway; 3 Institute of Marine Research, 5817 Bergen, Norway; 4 Helmholtz-Zentrum Geesthacht, Centre for Materials and Coastal Research, 21502 Geesthacht, Germany; 5 Centre for Ecological and Evolutionary Synthesis, Department of Biosciences, University of Oslo, 0316 Oslo, Norway; Woods Hole Oceanographic Institution, UNITED STATES

## Abstract

Predicting fish stock variations on interannual to decadal time scales is one of the major issues in fisheries science and management. Although the field of marine ecological predictions is still in its infancy, it is understood that a major source of multi-year predictability resides in the ocean. Here we show the first highly skilful long-term predictions of the commercially valuable Barents Sea cod stock. The 7-year predictions are based on the propagation of ocean temperature anomalies from the subpolar North Atlantic toward the Barents Sea, and the strong co-variability between these temperature anomalies and the cod stock. Retrospective predictions for the period 1957–2017 capture well multi-year to decadal variations in cod stock biomass, with cross-validated explained variance of over 60%. For lead times longer than one year the statistical long-term predictions show more skill than operational short-term predictions used in fisheries management and lagged persistence forecasts. Our results thus demonstrate the potential for ecosystem-based fisheries management, which could enable strategic planning on longer time scales. Future predictions show a gradual decline in the cod stock towards 2024.

## Introduction

**Fig 1:**
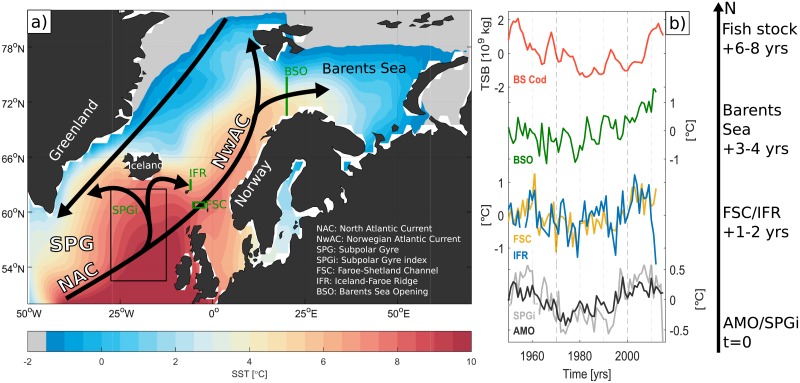
Barents Sea cod stock and upstream hydrography. (a) Winter sea surface temperature [28] and schematic of the major ocean currents in and between the subpolar North Atlantic Ocean and the Nordic Seas. Abbreviations are defined in the inset legend. (b) Time series of observed Barents Sea cod stock (total stock biomass; TSB) and temperature anomalies from the Nordic Seas Atlas [29] along the Atlantic water pathways used as upstream hydrographic predictors. The AMO and SPG indexes are defined in Material and methods. All anomalies are relative to 1950–2012. The average travel times of observed hydrographic anomalies from the subpolar North Atlantic (represented by AMO/SPGi) to the Nordic Seas (FSC/IFR) and to the Barents Sea, and their lagged influence on the cod stock, are indicated (cf. Table 1).

Seasonal to decadal predictions of fish stocks can provide valuable information for the management of marine resources. However, fish stocks are affected by both management and environmental conditions [1], and therefore inherently difficult to predict. Nevertheless, some Northeast Atlantic fish stock are potentially predictable because they are strongly associated with predictable environmental factors, such as multi-year to decadal variations in ocean temperature [2–8]. The ability to predict hydrography has recently been shown to be particularly high along the Atlantic water pathway, into the Nordic Seas and Barents Sea [9–11] (Fig 1a). Translating these predictions of the physical environment into ecological forecast products has, however, been little explored [12–14].

The Barents Sea is among the most biologically productive oceans in the world [15] and an economically important fisheries area. The major commercial stock is Barents Sea cod (*Gadus morhua*) [16], also referred to as Northeast Arctic cod. The Barents Sea ecosystem has been shown to be highly structured by the physical environment [16], which in turn is largely controlled by the inflow of Atlantic water (Fig 1a) [17, 18]. Several studies have accordingly related cod recruitment and cod stock variability in the Barents Sea to changes in the Atlantic water inflow. The strength and temperature of the Atlantic water inflow influence fish stocks directly and indirectly by affecting the size of the exploitable feeding area, the food supply, and the growth conditions [1, 2, 4, 16, 19–25]. Consequently, predictions of the Atlantic water inflow can be potentially used to assess future changes in the Barents Sea cod stock.

A particular potential for predicting the future development of the Barents Sea cod stock lies in the northward propagation of temperature anomalies from the subpolar North Atlantic toward the Arctic, with an associated travel time of 2–4 years [11, 26]. These anomalies have been shown to have a predictable impact on ocean temperatures and sea ice conditions in the Nordic and Barents Seas region [10, 11].

Here we show that Barents Sea cod stock variations can be predicted 7 years in advance, by combining the connectivity between the North Atlantic and Barents Sea with the strong co-variability between Barents Sea temperature and cod stock. This prediction horizon is substantially longer than that applied by previous prediction studies [21, 22, 27]. Despite its simplicity, our long-term prediction shows comparable skill to that of historical operational short-term predictions provided annually by the International Council for the Exploration of the Sea’s Arctic Fisheries Working Group (ICES AFWG). This highlights the possibility of fisheries management advice on a longer time scale than current practice. The influence of changes in fishing pressure on cod stock variability and predictions is also discussed.

## Materials and methods

### Biological data

Recruitment (REC3; number of 3-year olds) and total stock biomass (TSB; age 3 and older) for Barents Sea cod between 1950 and 2017 are taken from the latest report of ICES AFWG [30]. TSB is calculated using virtual population analysis (VPA), which means that the stock biomass is back-calculated based on the knowledge of death rates including fisheries and natural mortality sources. The TSB provided by ICES AFWG will be referred to as observations. It should be noted that when using VPA, adding another year of data also leads to a revision of the stock size some years back in time. For an illustration of the recent uncertainties in the stock estimates see the latest ICES advice for this stock (http://www.ices.dk). To obtain a relative measure of fishing efforts, the harvest rate of Barents Sea cod was calculated by dividing the annual catches (provided by ICES) by the total stock biomass.

ICES predictions are taken from the annual ICES AFWG reports since 1981, and are those which are the basis for the quota advice. Before 1981 such predictions were generally not provided. The length of predictions given in the reports vary from 2 to 6 years. Here we consider predictions 1–3 years ahead. In most years, predictions are given for several different exploitation rates. For each year, we have chosen the predictions which correspond to catch in tonnes (i.e., human impact) closest to the actual catches for the first three years in the prediction. Note that the 1987 assessment and advice was revised in mid-year (spring 1988), but that the figures from that revision are not shown. To evaluate the ICES predictions we construct a time series for each lead time and correlate it with the observation-based time series. Associated uncertainty estimates were calculated by sub-sampling 80% of the time series 1000 times.

### Oceanographic data

We use the Nordic Seas Atlas (NSA) [29] to obtain annual time series of observed temperature and salinity from the Atlantic inflow to the Nordic Seas between 1950 and 2012. The NSA contains data on a 0.25° grid at 29 vertical levels, which allows for a good representation of Atlantic water properties. The NSA is designed specifically for the region of interest and utilizes the extensive observations from the Nordic Seas and Barents Sea [29]. Time series of annual temperature and salinity are obtained along the Atlantic water pathways (Fig 1a); Faroe–Shetland Channel (FSC; 60.5–61°N, 2.5–5°W), Iceland–Faroe ridge (IFR; 62.5–63.5°N, 6°W) and the Fugløya-Bear Island section (Barents Sea Opening: BSO; 70–74°N, 20°E). The time series are constructed by averaging over the different areas and between 50–200 m depth, the latter commonly used to define the depth of Atlantic water [31]. The time series from FSC and IFR are also sometimes combined to create an average temperature and salinity of the Atlantic inflow across the Greenland-Scotland ridge, denoted *TS*_gsr_. As the NSA only provides data up to 2012, the hydrography from Nordic Seas Atlas is supplemented by observations from the ICES report on ocean climate (IROC), where observations of the northward flowing Atlantic water in the FSC are available for the time period 1950–2017 [32].

We also use North Atlantic climate indexes as potential predictors for Barents Sea cod stock. The Atlantic Multidecadal Oscillation (AMO) index [33] is defined as the annual (unfiltered) sea surface temperature (SST) between 0–70°N and was obtained from NOAA at http://www.esrl.noaa.gov/psd/data/timeseries/AMO, whereas the subpolar gyre index (SPGi) is defined as the annual mean SST (from HadISST [28]) between 52.5–62.5°N, 27.5-12.5°W [34]. Although the AMO index by definition reflects basin scale ocean temperature variations in the North Atlantic, the primary center of action is found in the subpolar North Atlantic [35]. The SPGi and AMO thus display similar variability (Fig 1b).

### Linear regression models

Predictions of the Barents Sea cod stock are obtained from multiple linear regression models of the form: *y*_*i*_ = *α*_0_ + *α*_1_*x*_*i*−*l*,1_ + … + *α*_*n*_*x*_*i*−*l*,*n*_ + *ϵ*_*i*_, where *y*_*i*_ is the predictand for each year *i*, *x*_*i*−*l*,*n*_ are the *n* predictor variables leading the predictand by *l* years, *α*_*n*_ are the regression coefficients, and *ϵ*_*i*_ is the residual term. The lag between predictors and predictands is based on the lagged correlation analysis presented in Table 1. The statistical validity of the regression equations was assessed by verifying that the residuals have constant variance, are independent, and are normally distributed. The regression models were also calculated after the linear dependence among the various predictors was removed to make sure that multicollinearity, i.e., correlated predictors, does not influence the regression models.

**Table 1:** Lagged peak correlations.

	AMO	SPGi	FSC		IFR		BSO	
	*T*	*T*	*T*	*S*	*T*	*S*	*T*	*S*
FSC	0.62(3)	0.75(3)	–	–	–	–	–	–
IFR	0.35(3)	0.28(3)	–	–	–	–	–	–
BSO	0.60(4)	0.52(4)	0.50(3)	0.77(2)	0.42(3)	0.59(2)	–	–
Cod	0.65(7)	0.54(7)*	0.57(7)	0.59(7)	0.45(7)	0.60(7)	0.56(2)	0.52(1)

Maximum lagged correlation between temperature (*T*) and salinity (*S*) time series along the Atlantic water pathway toward the Barents Sea, as well as their correlation with the Barents Sea cod total stock biomass between 1950 and 2012. Time lags (in parenthesis; unit: years) are given relative to the indexes in the top row, i.e., FSC temperatures lag the AMO index by 3 years. Correlations were calculated using detrended annual time series. Asterisk refers to correlations not significant at the 95% confidence level. Hydrography time series are based on the Nordic Seas Atlas [29]. AMO: Atlantic Multidecadal Oscillation; SPGi: Subpolar Gyre Index; FSC: Faroe–Shetland Channel; IFR: Iceland–Faroe Ridge; BSO: Barents Sea Opening.

A cross-validation method is applied to assess the statistical robustness of the regression models. Cross-validation is a statistical method used to reduce the problem of artificial skill produced by overfitting of random variability in a relatively short data record [36]. Cross validation is a resampling technique, where the available data are repeatedly divided in validation and verification data subsets. Specifically, for our analysis we first randomly select 80% of the data to construct the regression models (fitting period), which are thereafter used to predict the remaining 20% (prediction period) [37, 38].

Prediction skill is herein defined in terms of anomaly correlation coefficients and Brier scores. The correlation between observations and predictions and the Brier score (*β*) [36] are calculated for both the fitting period and the prediction period to assess the performance of the predictions. The Brier score is defined as $\beta =1-\sigma_{e}^{2}/\sigma_{o}^{2}$, where *σ*_*e*_ and *σ*_*o*_ are the variances of the error term and predictands, respectively. For predictions with small errors *β* will approach 1, whereas *β* = 0 indicates that the error of the model is the same as the variance of the predictand. The skill of the prediction models is compared with a random chance (RC) model, which is constructed by randomly shuffling the predictors, thus suppressing the relationship between the predictors and predictands. For significance testing a Monte Carlo method is applied where both the fitting period and the construction of RC are repeated 1000 times. Correlations and *β* are calculated for each iteration, and the median values and inter-quartile spread are thereafter used in the evaluation of the statistical predictions. We also compare the prediction skill against that of persistence forecasts. A lag-1 persistence forecast assumes that the cod stock in year *i* will repeat the observed value of the year prior to the forecast (*i* − 1). Similarly, a lag-*l* persistence forecast uses the observed value in year *i* − *l*.

For TSB predictions, Durbin-Watson statistic [36] reveals a statistically significant order-1 autocorrelation in the residual term (*r* = 0.4; no significant correlations at longer lags) if the regression model is trained and tested on the full time series. However, the random selection of data in the cross-validation procedure limits the serial correlation in the predictors and predictands, and, hence, in the residuals. The Durbin-Watson scores for the 1000 prediction periods suggest that autocorrelation is not a problem [36]. As a simple test, the regression models were also constructed using every second data point, hence effectively removing the lag-1 autocorrelation, and this also yields virtually the same regression coefficients. As the regression coefficients are robust with respect to the cross-validation procedure, autocorrelation is not explicitly accounted for in the regression models or in the error statistics. The statistical significance of correlations is nevertheless assessed according to a random phase test that takes autocorrelation into account [39].

## Results

### Upstream hydrographic influence on Barents Sea cod stock

The lagged peak correlations between hydrographic time series along the Atlantic water pathway (Fig 1b) and the Barents Sea cod stock (total stock biomass; TSB) are summarized in Table 1 (note that correlations are most often also significant 1 year before and after the peak lag). As previously reported, higher temperatures in the western Barents Sea (BSO) correspond to higher cod stock biomass with a lag of 2–3 years [1, 21]. Temperature variations in the Atlantic inflow across the Greenland–Scotland ridge (FSC and IFR) precede Barents Sea temperatures by 2–4 years, and, consistently, cod stock biomass by 7 years. The correlations and lags between salinity time series, and between salinity and cod stock, are comparable to those inferred from temperature (Table 1), suggesting that the predictive link between the subpolar North Atlantic and Barents Sea is of advective origin and related to Atlantic water circulation changes.

The Atlantic inflow to the Nordic Seas has been associated with variations in the strength and shape of the SPG [40]. This is reflected in a 2–3-year lag between the SPG index and Atlantic inflow temperatures (Table 1), and a 4-year lag between SPG and BSO temperatures. The correlations and lags are similar if we consider the AMO index, which reflects basin-scale ocean temperature variations in the North Atlantic [33]. There is a slight tendency for cod stock biomass to be more related to the upstream AMO index than the more local time series (BSO). This might be because the fish stock tends to be more influenced by lower frequency variability better captured by area-averaged indexes. In summary, observed hydrographic variability in the subpolar North Atlantic and in the Atlantic inflow branches to the Nordic Seas leads cod stock variations in the Barents Sea by approximately 6–8 years.

There is also a significant correlation between upstream hydrographic variability and cod recruitment. Consistent with the 7-year lag between upstream hydrography and total stock biomass, the maximum correlation between recruitment and hydrography in FSC and IFR is found for a 5-year lag (*r* ∼ 0.4), whereas recruitment leads stock changes by 2 years. The latter reflects that the contribution from a year-class to TSB becomes noticeable from the age of 4-5 years.

### Climate based cod stock predictions

Based on the identified lagged correlations between upstream hydrography and Barents Sea cod stock (TSB; Table 1), prediction models with a 7-year prediction horizon are constructed. The following predictor variables were considered, individually and in combination, and all leading cod stock TSB by 7 years: SPG and AMO indexes, and temperature and salinity from FSC and IFR (denoted *TS*_gsr_ when combined). We note that the aim of the statistical prediction models is not to perfectly model cod stock variability using all available information (including for example fishing mortality or TSB in previous years), but to assess the predictive ability of upstream hydrographic anomalies. Moreover, TSB or fishing mortality do not add predictive information on the 7-year prediction horizon assessed here.

**Fig 2:**
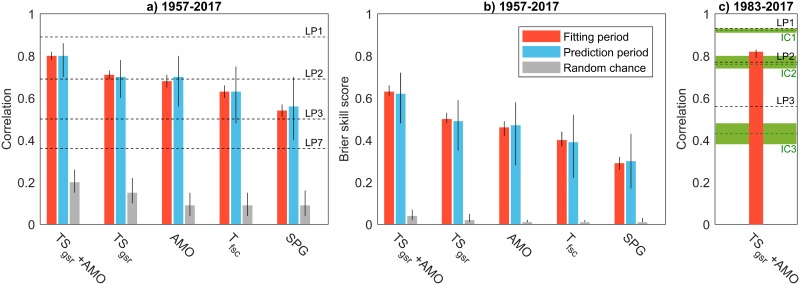
Evaluation of hydrographic predictors. (a,b) Prediction skill for the different cross-validated multiple linear regression models used to predict the Barents Sea cod stock biomass (TSB). The different hydrographic predictors are indicated on the x-axes (see Materials and methods for definitions). The bars show the median value from the cross-validation procedure, whereas the vertical black lines show the inter-quartile range. The horizontal black dashed lines are the lagged persistence (LP) forecasts at different lags, i.e., LP7 is the 7-year lagged persistence forecast. See text for abbreviations. (c) A skill comparison between our 7-year prediction and short-term predictions provided by ICES AFWG (1–3 years, abbreviated IC1–3; green dashed lines) for the time period 1983–2017. The green shading is the inter-quartile range obtained by sub-sampling of the data (see Data and Methods).

**Fig 3:**
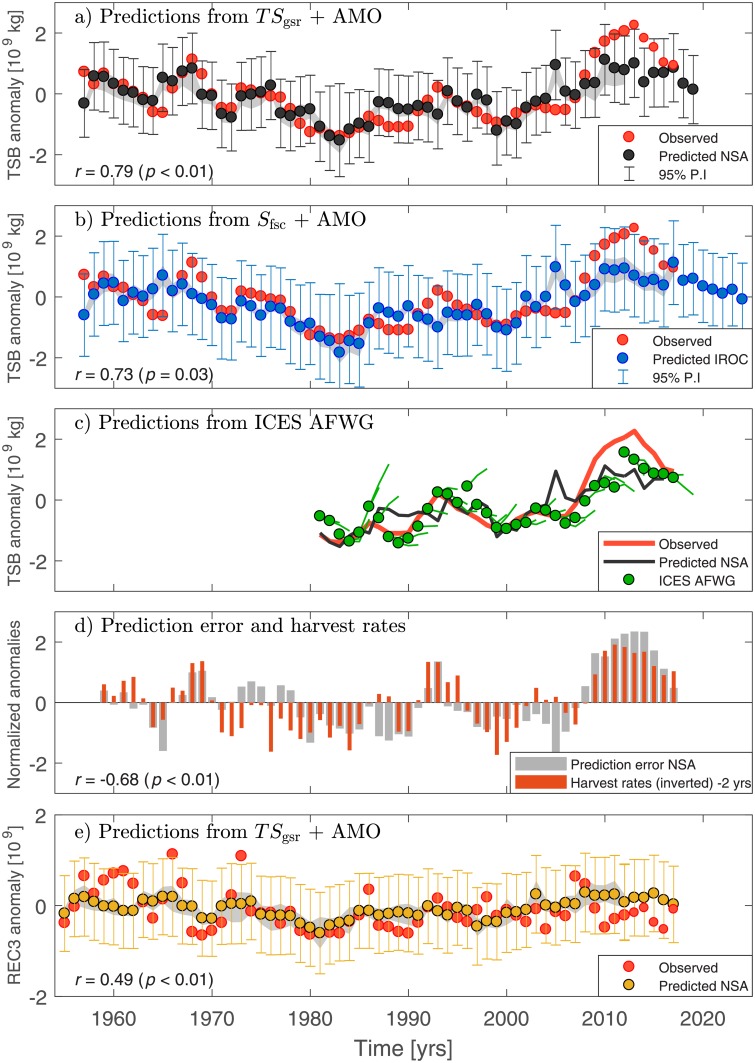
Predicted and observed Barents Sea cod stock. (a) Predictions of Barents Sea cod total stock biomass (TSB) anomalies based on the Atlantic inflow across the Greenland–Scotland ridge (*TS*_gsr_) from the Nordic Seas Atlas (NSA) and the AMO index 7 years in advance. (b) Predictions based on the AMO index and Faroe–Shetland Channel salinities (*S*_fsc_) from IROC. (c) Short-term predictions from the ICES AFWG compared with observations and the 7-year predictions from (a). ICES predictions are presented for 1–3 years, the first year indicated by the green circle. (d) Prediction error (observed minus 7-year NSA prediction) and harvest rate anomalies for Barents Sea cod. (e) Prediction of cod recruitment anomalies (REC3; number of 3-year olds) 5 years in advance. The vertical error bars in a,b,e show the 95% prediction interval (P.I), whereas the gray shading shows the spread in the predictions from the cross-validation procedure. All anomalies are relative to 1950–2012.

The temperature and salinity of the Atlantic inflow to the Nordic Seas are good predictors of cod stock TSB, but the skill is further increased if the AMO index is included (*r* = 0.79; Fig 2a and 2b). Prediction skill is lowest if the prediction model is based only on SPG temperatures. Skill computed for fitting and independent periods are similar for all models, suggesting that prediction skill is real and not artificial. The robustness of the regression coefficients is further illustrated by the limited spread of the different predictions from the cross-validation procedure (gray shading in Fig 3a and 3b). The TSB time series is significantly autocorrelated at lead times of 1–2 years, and lagged persistence forecasts accordingly show skill at short lead times. The skill of our predictions nevertheless outperforms that of lagged persistence forecasts for lead times longer than 1 year (Fig 2a and 2b; the persistence forecast corresponds to the observed value of the year prior to the forecast).

The future predictions based on hydrographic data from the Nordic Seas Atlas show a slight decline in cod stock toward year 2019, although values are still above the long-term (1950–2012) average. Using observed Atlantic water salinities from the Faroe–Shetland Channel (IROC) between 1957–2017 and the AMO index as predictors the correlation between observations and retrospective predictions is 0.73. Future predictions show a further decline in the cod stock toward 2024 (Fig 3b).

Predictions using the identified 5-year lag between hydrography and cod recruitment show some success in terms of correlations (*r* = 0.49; Fig 3e), but are less successful in reproducing the interannual variance (as measured by the Brier score; *β* = 0.27). The larger uncertainty in the predictions is also seen from the wider spread in the cross-validated regression coefficients (gray shading). Nevertheless, our predictions are more skilful than that of random chance and lagged persistence forecasts for any lead times (not shown).

### A comparison to operational predictions

Our predictions of TSB are now compared with short-term (1–3 years) predictions provided annually by the ICES AFWG (Fig 3c), and which takes into account both survey indexes, physical environment and prey abundance in predictions of recruitment [30]. The comparison is done for the time period 1983–2017 when ICES predictions are available. The ICES predictions with a 1-year lead time are better than our 7-year predictions, but do not outperform the lagged persistence forecasts (Fig 2c). For 2-year and 3-year lead times the ICES AFWG prediction skill is less than that achieved by considering upstream SST at a longer lead time. Unlike the ICES predictions, our long-term prediction also beats the persistence benchmark for lead times longer than one year. We note that the skill of our prediction model for the time period 1983–2017 (Fig 2c) is the same if the statistical model is trained only with data prior to 1983. The predicted cod stock anomalies from ICES AFWG also show a stock decrease for 2018 and 2019 with similar magnitudes as our long-term predictions (Fig 3c).

### Influence of fishing pressure

In addition to environmental factors, fishing exerts a major influence on Barents Sea fish populations [1, 23]. The statistical prediction models do not include explicit information on fishing pressure. However, as the statistical predictions are trained on historical cod stock data, fishing pressure and its influence on cod stock development is implicitly included in the models. To assess the influence of changes in fishing pressure on our cod stock predictions we compare the prediction error (observed minus predicted) with cod harvest rate anomalies (Fig 3d). It is seen that periods of persistent underestimation by our model (positive error) often correspond to low harvest rates (*r* = −0.68), harvest anomalies leading by 2 years. The zero-lag correlation is -0.47. A negative harvest rate anomaly thus results in a positive stock biomass anomaly the following years, which leads to an underestimated prediction.

## Discussion and conclusions

The skill of the simple statistical prediction models supports a dominant hydrographic influence on Barents Sea cod stock variability [2–4, 16, 19, 21, 22, 24]. Retrospective predictions of the cod stock capture much of the observed interannual variability (Fig 3a), but are particularly successful in capturing the multi-year to decadal stock fluctuations. Prediction skill on predominantly multi-year time scales is evident by low correlations between high-pass filtered (5-year cut-off) predictions and observations. The multi-year skill is consistent with previous studies finding low-frequency fish stock variability in the subpolar North Atlantic and Nordic Seas to be associated with hydrographic variability [3, 5, 6, 41].

Ocean temperature anomalies affect the cod stock in several ways, including through recruitment, individual growth, and predator and prey abundance [1, 2, 4, 16, 19, 21–24, 42]. The statistical relationship identified here represents the sum of all these, and, although the importance of specific mechanisms cannot be ascertained, we will in the following elaborate on some of them.

Starting at birth, the total larval mortality decreases when temperatures increases as the development time for cod eggs—from when they spawn to when they hatch—decreases, and, consequently, the accumulated time the larvae spend in the early and most vulnerable life stages is reduced [43]. Within the range of temperatures experienced in the Barents Sea, temperature furthermore has a significant positive effect on growth rates of cod [20, 44]. Another direct effect of increased ocean temperatures is an increase in the suitable feeding area, which offer release from food competition and cannibalism through extended overlap with prey and better adult stock productivity [1].

Indirectly, temperature affects early life stages and recruitment processes of the Barents Sea cod in complex ways, predominantly through grazing on the zooplankton species *Calanus finmarchicus* [45, 46]. Ocean temperature and the abundance of *C. finmarchicus* in the Barents Sea are linked by their mutual dependence on the inflow of Atlantic water from the Norwegian Sea; a strong inflow associated with increased ocean temperatures and increased advection of zooplankton from upstream production regions [43, 46]. In addition, as the ocean temperature increases and the sea ice extent is reduced the annual net primary production increases, leading to increased biomass and production of zooplankton and, hence, a larger fish stock [25, 47]. Primary production has accordingly been used in prediction models of cod recruitment in the Barents Sea [22].

Although lower than for cod stock biomass, the skill in predicting cod recruitment is similar to that obtained by [21] for the period 1947–1995 using ocean temperatures in the Barents Sea (Kola section) 2 years in advance as predictor. A stronger and more predictable relationship between hydrography and total stock biomass than with recruitment (cf. Fig 3) is consistent with total stock biomass variability reflecting the integrated (multi-year) effects on fish stocks, and, hence, being less sensitive to year-to-year differences in early life-history dynamics [48]. The total cod stock biomass furthermore mainly reflects adult cod dynamics. The adult cod population is most sensitive to changes in the extent of the suitable feeding area [1, 49], which closely corresponds to the size of the Atlantic water domain [4]. The size of the Atlantic domain, i.e., the ice-free area of the Barents Sea, is in turn largely a delayed response to the inflowing Atlantic water [17], consistent with temperature-driven cod distribution and population changes [1, 4].

In addition to hydrography, it has also been widely attempted to relate ecosystem variability in the Nordic and Barents Seas, including fish stocks, to large-scale atmospheric circulation [50] and in particular to the North Atlantic Oscillation (NAO), the dominant mode of atmospheric variability in the North Atlantic region [51]. However, the correlation between the winter NAO index [51] and the Barents Sea cod stock biomass is low and not significant (for all lead times) for the time period considered here. The NAO is also considered to be largely unpredictable for lead times longer than a year [52] and therefore cannot be used for multi-year predictions as presented here.

A major source of uncertainty in statistical predictions is whether the identified relationships hold when projected into the future [53, 54]. Changes in fishing pressure can for example lead to a mismatch between predicted and observed cod stock (Fig 3d). After the introduction of a harvest control rule for cod in 2003 and the subsequent introduction of measures to avoid underreporting of catches, the harvest rates stabilized at a low level from 2007 onwards [1, 30]. The harvest rates are expected to remain stable in the future [30], but as any changes in fishing pressure can lead to prediction biases, different levels of fishing pressure should be considered when further developing predictions for cod stock biomass.

In conclusion, we have demonstrated that a significant part of future changes in the Barents Sea cod stock can be skilfully predicted based on upstream ocean variability. The 7-year prediction horizon is based on the poleward propagation of hydrographic anomalies along the Atlantic water pathway toward the Barents Sea (Fig 1). The specific temperature-fish stock relations that the predictions presented here are based on are specific to the Barents Sea cod, but the methodology applied should be applicable to other regions and stocks affected by delayed ocean climate signals. Dynamical prediction models have demonstrated particular multi-year forecast skill of ocean temperature in the subpolar North Atlantic [10]. Whether this ability to predict the ocean environment translates into predictable fish stock responses has not yet been much assessed, but there are indications that the skill extends to large marine ecosystems on the adjacent coastal shelves [7].

Both our statistical long-term predictions and the short-term ICES predictions show a gradual decline in the cod stock over the next few years. This is the opposite of the development expected over this century from future climate change projections. In a warming world, higher ocean temperatures and reduced sea ice extent may result in favorable conditions for increased biological production and a northward migration of fish stocks [55, 56]. This apparent discrepancy between decadal cod stock changes (captured by our predictions) and future projections results from large internal variability in the Barents Sea climate system, expressed by periods of decadal temperature decrease and sea ice growth superimposed on a gradual warming trend [11, 57]. Multi-year predictions, like those presented here, thus fill the gap between short-term operational predictions and century-scale climate change projections.

Integrating the identified relationship between upstream climate variability and cod stock into management frameworks could enable the possibility of better fisheries management advice on a longer time scale than today. Seasonal forecasts are already widely used in management, predominately informing on the spatial distribution of fish stocks [13, 14]. Similarly, multi-year to decadal forecasts can be used to inform stakeholders and decision makers on expected longer-term changes in fish stocks. For the Barents Sea specifically, the warming and loss of sea ice that took place between 2004 and 2012 could have been predicted [11, 58], and this information could have been used to predict the northward expansion of boreal fish species that occurred over the same period [4]. Such prior knowledge could enable the fisheries community to better survey, exploit, and manage these resources [59, 60]. However, we note that although this study has focused solely on cod, the management of fish stocks in the Barents Sea needs to have a multi-species perspective [61].
